# Long-lived *Temnothorax* ant queens switch from investment in immunity to antioxidant production with age

**DOI:** 10.1038/s41598-019-43796-1

**Published:** 2019-05-13

**Authors:** Matteo Antoine Negroni, Susanne Foitzik, Barbara Feldmeyer

**Affiliations:** 1 Institute of Organismic and Molecular Evolution, Johannes Gutenberg University of Mainz, Mainz, Germany; 2 Senckenberg Biodiversity and Climate Research Centre (SBiK-F), Molecular Ecology, Senckenberg, Frankfurt Germany

**Keywords:** Ecology, Evolution, Transcriptomics

## Abstract

Senescence is manifested by an increase in molecular damage and a deterioration of biological functions with age. In most organisms, body maintenance is traded-off with reproduction. This negative relationship between longevity and fecundity is also evident on the molecular level. Exempt from this negative trait association, social insect queens are both extremely long-lived and highly fecund. Here, we study changes in gene expression with age and fecundity in ant queens to understand the molecular basis of their long lifespan. We analyse tissue-specific gene expression in young founding queens and old fecund queens of the ant *Temnothorax rugatulus*. More genes altered their expression with age in the fat body than in the brain. Despite strong differences in ovary development, few fecundity genes were differentially expressed. Young founding queens invested in immunity (*i*.*e*. activation of *Toll* signalling pathway) and resistance against environmental and physiological stress (*i*.*e*. down-regulation of TOR pathway). Conversely, established older queens invested into anti-aging mechanisms through an overproduction of antioxidants (*i*.*e*. *upregulation of catalase*, *superoxide dismutase*). Finally, we identified candidate genes and pathways, potentially involved in the association between fertility and longevity in social insects and its proximate basis.

## Introduction

Senescence occurs in almost all organisms, despite the fact that it increases intrinsic mortality^1^. Understanding the origin of this evolutionary puzzle is a fundamental, but challenging question in biology. Evolution of senescence is considered to result from a decline in strength of selection with age, due to extrinsic mortality (e.g. predation, starvation, diseases, accident), and/or the costs of body maintenance^2,3^. Evolutionary theories of aging regard senescence as a consequence of deleterious mutations expressed late in life due to antagonistic pleiotropy or mutation accumulation^3^. Physiologically, senescence is characterised by a general decline in biological functions including immunity and homeostasis^4^. At the molecular level, aging is commonly considered as the result of the progressive accumulation of molecular damage due to inadequate somatic repair^3–5^. Causes of molecular damage include metabolically generated radical oxygen species (ROS), spontaneous biochemical errors in replication, transcription, translation or maturation, and environmental factors such as toxic component or extreme temperatures^4,6^. The oxidative stress theory points to the accumulation of oxidative damage as the main proximate cause of aging^4^, which implies that long-lived organisms should be characterized by: (i) better molecular repair abilities, (ii) lower rates of molecular damage (i.e. lower production of ROS or reduced replication mistakes); (iii) higher production of anti-oxidants. Although being intensively studied, the highly complex biological process of aging is still poorly understood and many lifespan determining factors remain unidentified^5,7–9^.

Underlying the cost of body maintenance, lifespan appears to be commonly traded-off with reproduction^10^. Although this trade-off is considered widespread across the animal kingdom, there is an increasing number of examples of increased growth and reproduction through manipulation of diet composition, without a cost on lifespan challenging the trade-off hypothesis^11^. In particular, social insect queens, are apparently bypassing the common negative association between fecundity and longevity, as they are both, extremely long-lived and highly fecund compared to their workers^12,13^. In most eusocial species, phenotypic plasticity rather than genetic differences cause those tremendous life history differences between female castes, which develop from the same genomic background^14^. Hence, social insects offer the unique opportunity to study how differential gene expression regulates different rates of aging^15^.

Insect queens are well cared for by their workers, and the availability of ample resources could explain why queens can invest in both lifespan and reproduction. However, in many species, including humans, fruit flies, rodents and monkeys, dietary restrictions lengthen lifespan, while for instance in *Drosophila* a protein-rich diet reduces it^16,17^. This link between diet and lifespan involves the nutrient sensitive IIS and TOR pathways, which are conserved from yeast to humans^18^. The inhibition of those pathways through dietary restriction inhibits cell growth and had a negative effect on fecundity, but improves stress resistance. Those observations suggest that in social insects (i) certain pathways or biological processes may be differently regulated compared to *Drosophila*^19^, and/or (ii) that queens receive processed food from workers with a specific composition that modifies the effects of nutrients on lifespan^20,21^. In contrast to rodents and *Drosophila*, caloric restriction has a negative effect on both lifespan and reproduction in *Temnothorax rugatulus* queens (unpublished results).

The link between longevity and investment in immunity might also differ between social insects and other species. In *Cardiocondyla* ants, queens that activated their immune system are shorter lived^22^, whereas across *Caenorhabditis* species longevity and immunocompetence seem to be positively linked^23^. Recent studies focussing on gene expression differences between castes and variation with age point towards a complex link between molecular damage, repair mechanisms and lifespan, which are potentially species-specific, and suggest that investments in fecundity and body maintenance may shift during an ants’ life^5,24–26^.

**Figure 1:**
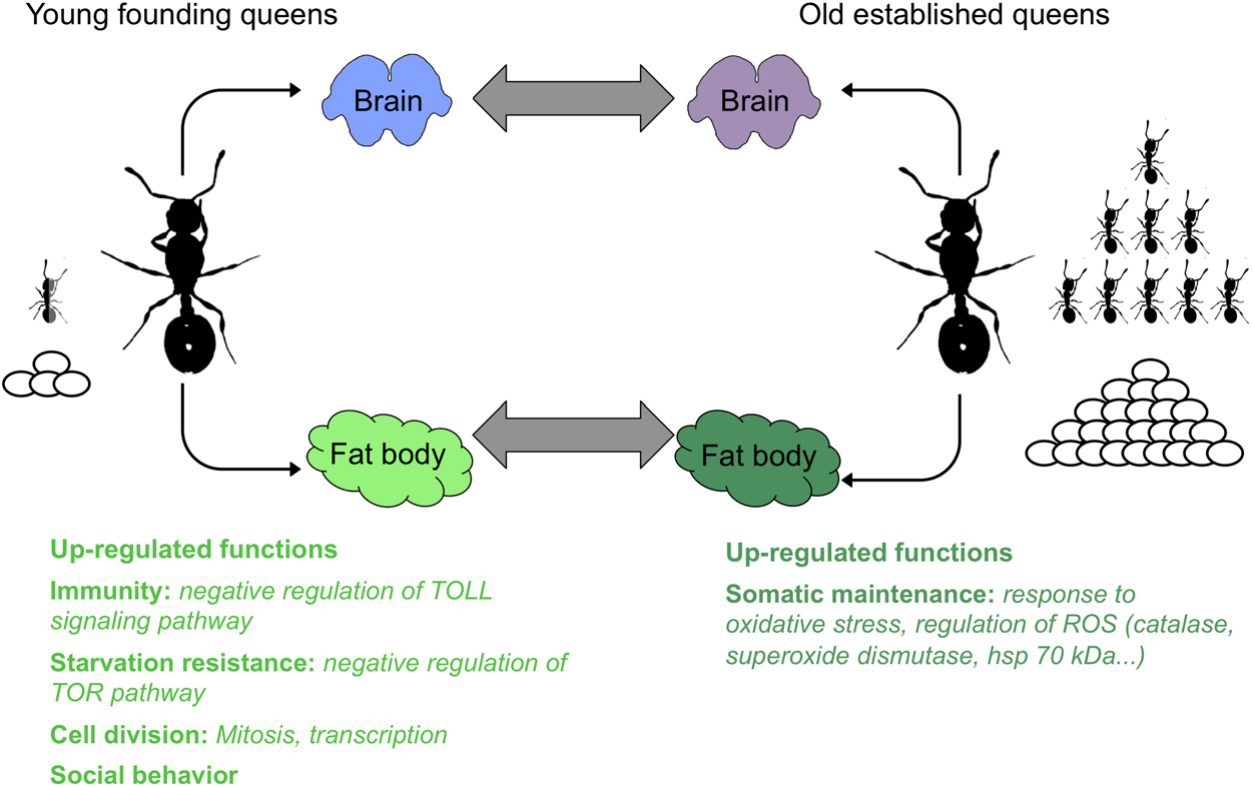
This figure illustrates the experimental design in our investigation of tissue-specific changes in gene expression with age and fecundity in ant queens. RNA was extracted from the brain and the fat body of N = 8 young founding queens and N = 8 old established queens and two different differential expression analysis were conducted within-tissues/between-age classes comparison (grey double arrows); The young founding queens were found in the field with 0 to 8 workers and had much less developed ovaries compared to the old established one for which the colony size ranged between 197 and 337 workers. The eggs illustrate relative fecundity of queens, and worker number illustrate colony size. The coloured boxes summarize the findings about differential investment in traits and function out of the comparison between young and old queen fat bodies (ROS = radical oxygen species).

Here, we compare tissue-specific gene expression between old queens (several years old) and young founding queens (a few weeks old) of the ant *T*. *rugatulus* (Fig. 1). Our aim was to investigate which genes and pathways change their expression over the lifetime of ant queens. We selected brain and fat body as focal tissues because (i) the brain is the production site of several physiological important hormones and is prone to numerous age-related degenerative diseases^27^, (ii) the fat body is a physiologically very active organ where most of the haemolymph proteins are synthesized and processed^28^.

Independent from age, we expected young queens to invest more into immunity and starvation resistance, as they were likely to be exposed to pathogens during the mating flight and consequent colony foundation, and are in the process of raising their first worker generation solely based on their body reserves. Conversely we predicted old queens, which experience a low extrinsic mortality as they are well cared for by their large workforce, both in terms of food provisioning and social immunity, to concentrate on fecundity and body maintenance mechanisms^29,30^.

## Material and Methods

### Field collection and ant maintenance

*Temnothorax rugatulus* is a small North American ant widely distributed throughout the western part of the continent. Colonies live in small crevices or under stones in high elevation oak and pine forests. Two queen morphs occur in this species: the large macrogynes and the small microgynes^31,32^. Here, we focussed on the large macrogynes that can start their colony independently and live mostly in monogynous societies. Queens of monogynous *Temnothorax* species can live more than a decade^12,33^.

Ant colonies were collected in August-September 2015 in the Chiricahua Mountains, Arizona at seven sites (coordinates: Supplementary Table S1), approximately 2–3 months after the mating flight starting in June^31^. Thus, founding queens were only 2–4 months old and had zero to eight minim workers (first generation workers; Table S1 ^33^). For the old queen cohort, we selected the largest monogynous colonies from our collections, which contained between 197–337 workers (Table S1). Based on their colony size and the slow growth rates of *Temnothorax* colonies, we estimated that these queens were at least several years if not a decade old^12,33^. We selected eight queens and their colonies from each of the two age categories (Fig. 1) and those 16 colonies were kept individually in artificial nests as described in^26^.

### Dissection, fertility measurements and RNA extraction

After 11 weeks under stable conditions (22 °C, 12L:12D), the 16 queens were killed by decapitation, and brain, fat body and ovaries were immediately dissected on ice. Tissue dissection took less than 10 min for each queen. Order of dissection was randomized over age classes to control for potential confounding effects. For each queen the brain and fat body were separately and individually homogenized in 50 μL of TRIZOL for storage at −20 °C before RNA extraction. RNA was extracted from fat body and brain samples separately using the RNeasy mini kit (Qiagen), resulting in total in 32 RNA samples (16 brains and 16 fat bodies, of 8 young and 8 old queens). Library preparation was conducted according to standard protocols at BGI Hongkong, which also sequenced 100 bp paired reads on an Illumina HiSeq2000/2500. The ovaries were further dissected and a photo was taken for fertility measurements (magnification x20; camera *Leica DFC425*; measurements: Leica software *LAS version 4*.*5*). To analyse differences in fecundity between age classes, we used a linear model with ovary length (in mm) as a dependant variable, and we used a generalized linear model with a *quasipoisson* distribution (link function = log; overdispersion = 1.6) with the number of maturing (white) eggs (count data) as a dependent variable. Age class was implemented as an explanatory variable in both models. Statistical analyses were conducted in R v. 3.0.2^34^.

### Gene expression analysis and annotation

After trimming the raw reads (filtering out the low quality reads coming from sequencing artefacts) with *Trimmomatic-v0*.*36*^35^, and quality checking using *FastQC-v0*.*11*.*5*^36^, all paired reads were *de novo* assembled. We used different assemblers, *Soap*^37^, *Bridger*^38^, *Trinity* (trinityrnaseq-Trinity-v2.4.0)^35^, and also constructed a meta-assembly of the three assemblies with *MIRA*^39^. Using the program *TransRate*^40^, we conducted a quality comparison between the four assemblies (assembly details and quality estimates in the Supplementary Table S2). Especially based on the contig length (set of overlapping sequences rebuilding a transcript sequence), and back-mapping rate (percent raw sequences mapping to the *de novo* assembled contigs), we decided to choose the Trinity assembly for further analysis (back mapping rate of 87%).

We used *RSEM*-v1.3.0 with the implemented *Bowtie2* aligner in order to obtain read count estimates per contig and sample. The read count consists of the number of sequence reads that align to a given contig, reflecting the expression level of the corresponding gene. In order to visualize sample similarity and detect putative outliers, we built hierarchical clustering dendrograms of samples based on the Euclidean distance (using *average* as agglomeration method, with the R command *htclust* from the package *stats*^41^), based on the entire dataset, but also on a subset only including brain or fat body data, respectively. The differential gene expression analysis performed with pairwise contrasts with the R package *Deseq2-v1*.*2*.*10* (*contrast* function). Venn diagrams were built using the online available tool Venny (http://bioinfogp.cnb.csic.es/tools/venny/*)*. Based on the top 15,000 contigs with the highest across sample variance in expression, we assessed the overall variance across samples within groups and compared old versus young queens (across all tissues), and brain versus fat body (across both age classes), using a permutational multivariate analysis of variance (PERMANOVA, 999 permutations) based on the Bray-Curtis similarity in the software Primer 6.0 & PERMANOVA (Primer-E Ltd.).

To annotate the contigs we conducted a *BlastX* homology search (based on alignment and sequence similarity)^42^ against the non-redundant invertebrate protein database (state June 2016). Nucleotide sequences were translated into amino-acid sequences with *Transdecoder-v3*.*0*.*1*^35^, before conducting the gene ontology (GO) and the Kyoto encyclopaedia of genes and genomes (KEGG) term annotation using *InterProScan-v5*.*25-64*.*0*^43^, assuming evolutionary conservatism of gene functions across species. For this within tissue, between age classes comparison, we furthermore manually annotated differentially expressed genes (FDR-p < 0.05), with Log2FoldChange >2 (at least four times more expressed in one age class relative to the other), using the *Uniprot* database (www.uniprot.ong), with *Drosophila* annotations when available, or other organism if not (*Drosophila*: 35.2%; other insects: 4.2%; other non-insects: 60.6%).

In order to check whether contigs showing the same age-specific expression pattern in the two tissue types (upregulated contigs in a given age class, both in brain and in fat body) were housekeeping genes, we made use of *Busco v*.*3*^44^ using the conserved insect database as backbone.

To identify gene networks, a weighted gene co-expression network analysis (WGCNA) was performed using the R package *WGCNA*^45^ on the top 15,000 contigs with the highest variance in expression across samples. For these analyses we used the following parameters soft thresholding power of 5 for brain and 10 for fat body data (following the manual instructions), with the minimal number of contigs per cluster set to 220 and a dissimilarity threshold of 0.2. No obvious outliers were detected from the clustering analysis of both tissues, so that all samples remained in the analysis (see dendrograms Supplementary Fig. S1a,b).

In order to investigate which biological processes were differentially activated in the two different tissues of each age group, we performed a GO term enrichment analyses based on the subsets of differentially expressed contigs using the R package *TopGo -v-3*.*6*^46^, with the “weight01” algorithm. This was done separately for contigs upregulated in the brain and fat body of old and young queens. A GO enrichment was also performed on the co-expression modules. The enrichment analysis revealed the over-representation of functional categories in our test group (the differentially expressed genes in young/old queens and fat body/brain) in comparison to the reference set (all contigs in the transcriptome). The p-values for each GO term were obtained by using a *Fishers exact* test. Additionally, we conducted a functional enrichment based on the list of unique isoforms (with duplicates filtered out based on the gene identity), which revealed qualitatively similar results compared the one performed on the entire list of differentially expressed contigs (see Supplementary Table S3).

## Results

### Fertility

**Figure 2:**
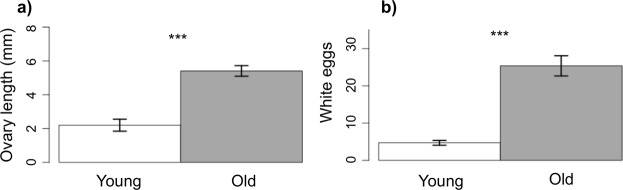
Young queens are less fecund than older queens. Differences in ovary development include (**a**) length of ovarioles and (**b**) the number of eggs in development. Old queens have significantly longer ovaries and a higher number of eggs than young ones (respectively: F_1_ = 46.23, p-value < **0.0001**; Chi2 = 74.7, p-value < **0.0001).**

Queens of the two age classes strongly differed in fecundity (Fig. 2a,b). Old queens had longer ovarioles (F_1_ = 46.23, p-value < 0.0001), and about five times more white eggs in their ovaries (X^2^_1_ = 74.7, p-value < 0.0001) compared to young queens.

### Transcriptome analyses

The *Trinity* assembly resulted in 128,764 number contigs, of which 39% were annotated with *BlastX* against the non-redundant insect database (Supplementary Table S4). The hierarchical sample clustering analysis revealed that gene expression differed more strongly between tissue types than between age classes (see dendrogram Supplementary Fig. S2, PCA plot Supplementary Fig. S3). However, old queens differed more in their gene expression than young queens (PERMANOVA, 999 permutations; t = 2.37, p-value = 0.018). Clustering by age classes were more pronounced in the fat body than in the brain (see dendrogram Supplementary Fig. S4a,b; and PCA plots Supplementary Fig. S5a,b). Moreover, the between-sample overall dispersion in gene expression was higher in the fat body compared to the brain (PERMANOVA, 999 permutations; t = 3.38, p-value = 0.012).

#### Differential gene expression

**Figure 3:**
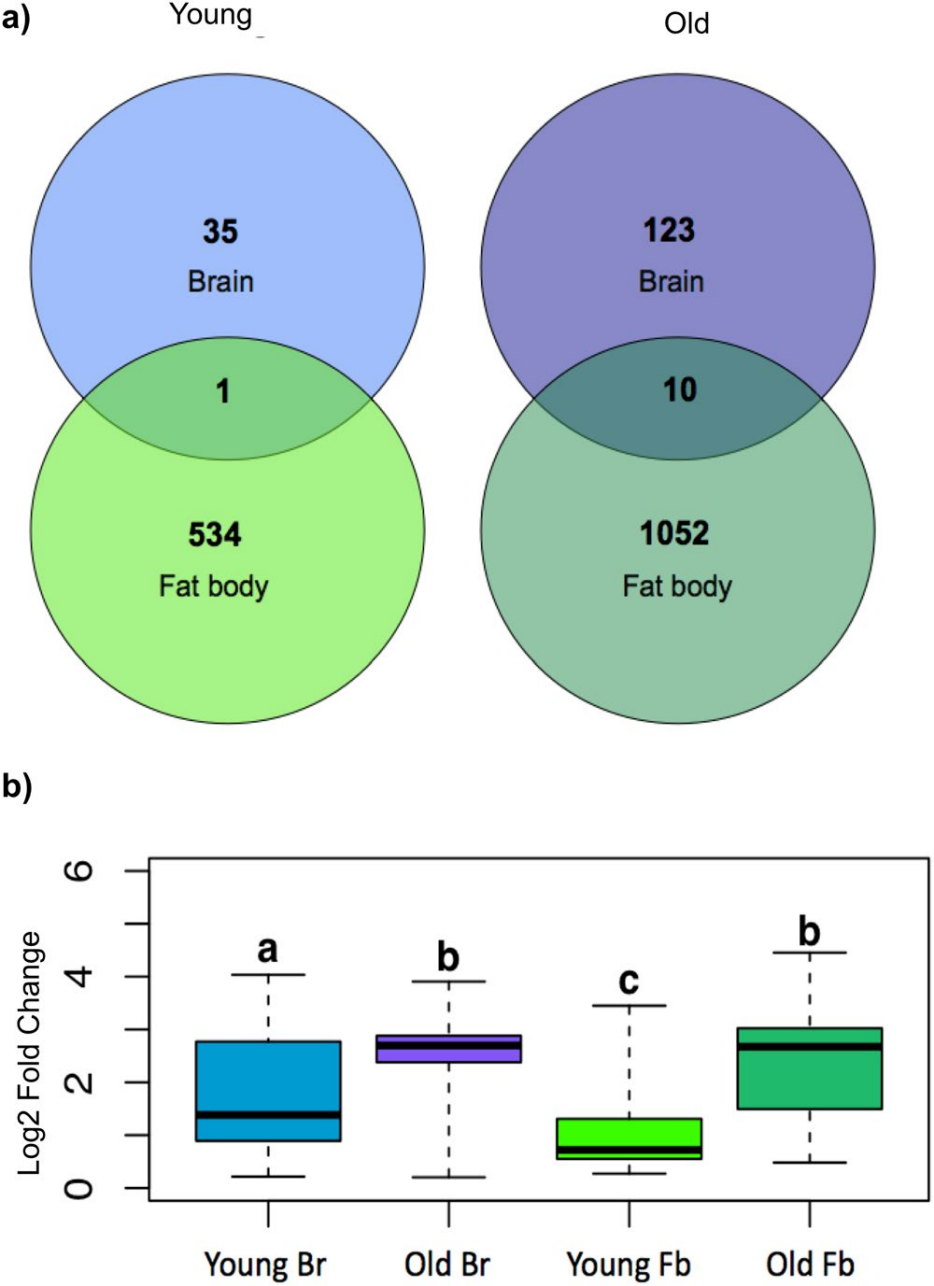
Summary of the within tissue differential gene expression analysis between old and young queens: (**a**) Venn diagrams depict the number of upregulated genes per tissue, between age classes with overlaps for contigs shared across tissues; (**b**) Relative expression level (Log2 Fold Change) of upregulated genes per tissue and age class. Upregulated contigs in young queen brains compared to old ones are in light blue; up-regulated contigs in old queen brains compared to young ones in purple; up regulated contigs in young queen fat bodies compared to old ones in light green; up regulated in old queen fat bodies compared to young ones in dark green. Test of the effect of tissue in interaction with age class reveled a significant effect of both tissue, age class and interaction (respectively: Chi2 = 9.77, Df = 1, p-value 0.0018; Chi2 = 509.85, Df = 1, p-value < 0.0001; Chi2 = 9.77, Df = 1, p-value 0.008), and the results from the post-hoc pair wise comparison are summarized with letters (at the threshold of 0.05 after Bonferroni correction).

Differential gene expression analysis per tissue and between age classes (Fig. 3a) revealed many more differentially expressed genes between old and young queens in the fat body compared to the brain (Pearson’s Chi^2^ test: X^2^ = 1154.6, df = 1, p-value < 0.0001; Fig. 3a), although the absolute LogFoldChanges were higher in the brain (Fig. 3b). In total, 1,597 contigs were differentially expressed in the fat body between the two age classes, compared to only 169 between the brain of young and old queens (FDR-p < 0.05, see Supplementary Tables S5 and S6 respectively for the brain and the fat body). Only few (N = 11) upregulated contigs were shared across tissues (Fig. 3a, and Supplementary Tables S5 and S6f,g, for old and young queens respectively), with one of them *40S ribosomal protein S2*, being involved in oogenesis in *D*. *melanogaster* and consistently upregulated in old queen fertile queens both for brain and fat body. Among these 11 overlapping contigs we found that two of them matched the conserved insect database (*Busco*) and might thus represent housekeeping genes (see Supplmentary Tables S5 and S6f,g). In both tissues, old queens upregulated many more genes than young ones (Pearson’s Chi^2^ test; brain: X^2^ = 55.7, df = 1, p-value < 0.0001; Pearson’s Chi^2^ test; fat body: X^2^ = 173.9, df = 1, p-value < 0.0001), and those genes also had a significantly higher change in expression (linear-mixed model: Chi2 = 509.85, Df = 1, p-value < 0.0001; Fig. 3b, read count per sample available online at NCBI’s Short Read Archive (SRA) under study accession N° GSE111415, see data accessibility section).

We identified interesting candidates among the differentially expressed genes involved in longevity or fecundity regulation, or associated with the social environment (Table 1). Indeed, we detected six contigs upregulated in old queens annotated to genes involved in longevity pathways of humans and *Drosophila*, and positively associated with lifespan (see KEGG database, *Longevity regulating pathway*). In old queens, *Superoxide dismutase*, and *heat shock* 70 *kDa protein cognate 4* showed a higher expression in both tissues, while *catalase* and *heat shock 70* *kDa protein IV* were specifically upregulated in the fat body. These genes play a major role in the regulation of oxidative stress and protection against reactive oxygen species (*ROS*)^47–50^. Surprisingly, only few contigs associated with fertility were detected among the age-specific genes expressed in both tissues (2% in the fat body versus 3% in the brain, LogFoldChange >2, FDR-p < 0.05). No obvious candidates were discovered in the pathway analysis (Supplementary Table S7).

**Table 1:** Candidate genes up-regulated per tissue and age class comparing young and old queens.

Age	Tissue	Blast		LFC	P-val	Uniprot	
		Annotation	Species			Function	Species
Young queens	Fat body	collagen alpha-1(IV) chain	*Trachymyrmex septentrionalis*	2.37	0.011	Oviduct morphogenesis	*D*. *melanogaster*
		alkylated DNA repair protein alkB	*Solenopsis invicta*	0.48	0.003	DNA repair	*Homo sapiens*
		mitotic spindle assembly checkpoint protein MAD1	*Wasmannia auropunctata*	0.47	0.023	Sister chromatin cohesion	*Homo sapiens*
Old queens	Fat body	catalase isoform X2	*Metaseiulus occidentalis*	4.62	0.001	Determination of adult lifespan	*D*. *melanogaster*
		heat shock 70 kDa protein IV	*Strongylocentrotus purpuratus*	4.21	0.004	Negative regulation of apoptotic process	*Homo sapiens*
		heat shock 70 kDa protein cognate 4-like	*Polistes dominula*	4.14	<0.001	Cellular response to topologically incorrect protein	*D*. *melanogaster*
		heat shock 70 kDa protein 4-like	*Parasteatoda tepidariorum*	3.68	0.017	Negative regulation of apoptosis	*Homo sapiens*
		superoxide dismutase Cu-Zn	*Athalia rosae*	3.60	0.022	Age-dependent response to oxidative stress	*D*. *melanogaster*
		catalase-like	*Trichogramma pretiosum*	3.49	0.028	Determination of adult lifespan	*D*. *melanogaster*
		40S ribosomal protein S2	*Copidosoma floridanum*	3.36	0.035	Oogenesis	*D*. *melanogaster*
		heat shock 70 kDa protein cognate 4	*Trachymyrmex cornetzi*	3.13	0.005	Cellular response to topologically incorrect protein	*D*. *melanogaster*
		translationally-controlled tumor protein	*Apis cerana*	2.71	0.029	DNA repair	*D*. *melanogaster*
		ataxin-2 homolog	*Solenopsis invicta*	2.81	0.038	Oocyte differentiation	*D*. *melanogaster*
	Brain	superoxide dismutase Cu-Zn-like	*Rhagoletis zephyria*	2.68	0.04	Age-dependent response to oxidative stress	*D*. *melanogaster*
		*C*. *briggsae* CBR-SODH-1 protein	*Caenorhabditis briggsae*	3.32	<0.001	Defence response to Gram-positive bacterium	*Caenorhabditis briggsae*
		heat shock 70 kDa protein cognate 4	*Linepithema humile*	3.09	<0.001	Cellular response to topologically incorrect protein	*D*. *melanogaster*
		40S ribosomal protein S2	*Copidosoma floridanum*	3.21	0.003	Oogenesis	*D*. *melanogaster*

We list only the genes likely involved in the regulation of longevity and/or fecundity or associated with the social environment (see the entire list of differentially expressed genes between old and young queens in the brain and in the fat body respectively Tables S4 and S5). Shown are the blast annotation with corresponding species, the logFoldChange (LFC) from the comparison between old and young within tissue, the corresponding corrected P-value, the functional annotation made on UniProt as well as the corresponding species (*D*. *melanogaster* for *Drosophila melanogaster*).

#### Functional enrichment

The lists of significantly enriched functions in the fat body and the brain for each age class are presented in Supplementary Table S8 (Supplementary Table S9 summarizes the full results of the functional enrichment analysis).

In the list of enriched functions in upregulated genes in young queens’ fat bodies, we found the *Toll pathway*, which is known to play a major role in insect immunity^51^. Also the *negative regulation of the TOR pathway*, a universal nutrient sensitive pathway, could be explained by food deprivation of young queens with no or only few workers during the founding phase. Interestingly, *tryptophan catabolism into kynurenine*, which is negatively associated with lifespan in *Drosophila*, was also enriched in the fat bodies of young queens^52^. Finally, mechanisms ensuring DNA integrity seem to be activated in young queens’ fat bodies, such as *mitotic cell division checkpoint* or *sister chromatin cohesion*.

In old queen’s fat bodies we found functions associated with translation, but without any transcription or RNA degradation in contrast to the young queens, which may indicate a less intense protein turnover and a less dynamic protein composition. Interestingly, our results suggest an activation of mechanisms involved in regulation of oxidative damage such as *regulation of response to ROS* or *response to oxidative stress*. The upregulation of genes involved in the *S-adenosylmethionine biosynthetic process* was surprising as an increase of this molecule is linked to aging in *Drosophila melanogaster*^35^. Finally, the enrichment of *acetyl-CoA metabolism* in both tissues is also relevant as acetyl-CoA plays a role in the epigenetic regulation of stress response genes through histone acetylation^53^.

#### Weighted gene co-expression network analysis

Co-expression network analysis (WGCNA) for the fat body revealed that two, of the 15 modules were significantly positively associated with young age and none with old age. Of the 11 co-expression modules of the brain data, one was positively associated with young age and none with old age (Fig. S6a,b). Among modules positively associated with young age, we found a significant overrepresentation of genes with functions in *regulation of cell growth*, and *lipid catabolic process* in the fat body (module 6 and 7, df = 1, p < 0.0001; results on enriched functions per module for each tissue are presented Tables S10a and S10b respectively for brain and fat body).

## Discussion

With their extreme lifespan and seemingly circumventing the widespread trade-off between longevity and fecundity, social insect queens provide the unique opportunity to investigate the molecular basis of senescence. We studied tissue-specific changes in gene expression with age in queens of the ant *T*. *rugatulus*. Old and young queens strongly differed in ovary length, which was weakly reflected by a differential expression of fecundity-associated genes in the two tissues investigated. We show age-related changes in the expression of longevity and immunity genes and pathways, both in the brain and in the fat body (Fig. 3a). Moreover, we provide evidence for regulatory changes of candidate genes and pathways compared to *Drosophila* and other solitary organisms, making *T*. *rugatulus* a good candidate to investigate the atypical positive association between lifespan and reproduction in social insects (Table 2).

**Table 2:** List of candidate genes and pathways associated with longevity and potentially involved in the reshaping of the trade-off between lifespan and reproduction in social insects.

Candidate gene	Biological process	Trait association in solitary organisms	Age-related expression pattern in solitary organism	Age-related expression pattern in *T*. *rugatulus* queen
catalase isoform X2/ *catalase-like*	Reduction of oxidative stress (longevity pathway, mammals and *Drosophila*)	Positively associated with longevity in *Drosophila*^47^	Decreasing in the rat brain and in *Drosophila*^67,68^	Increasing in the fat body
*superoxide dismutase [Cu-Zn]*		Positively associated with longevity in *Drosophila*^47^	Decreasing in the rat brain^68^	Increasing in the fat body and in the brain
*SAM synthase-like*/ *SAM synthase isoform X5*/ *SAM synthase isoform X2*/ *SAM synthase isoform X1*/ *SAM synthase isoform X3*	SAM biosynthesis (SAM metabolism)	Negatively associated with longevity in *C*. *elegans*^73^ (SAM degradation extends lifespan in *Drosophila*^71^)	NA	Increasing in the fat body
*kynurenine/alpha-aminoadipate aminotransferase*, *mitochondrial-like*	Tryptophan catabolism into kynurenine (kynurenine pathway)	Positively associated with aging^52,75,76^ and negatively associated with longevity in *Drosophila*^74^	Increasing in human serum^52^	Decreasing in the fat body

Additional information comprises the according biological process, trait association in other organisms with reference as well as information on the respective expression pattern in the solitary species in comparison to *T*. *rugatulus*^59^.

By an order of magnitude more genes changed their expression with age in the fat body compared to the brain. Moreover, typical longevity genes such as heat shock proteins *70* *kDa* (*hsp70*), *superoxide dismutase* (*SOD*) or *catalase* (*CAT*)^54,55^ were found among the differentially expressed genes in the fat body in higher number compared to the brain, suggesting that aging or the fight against senescence rather takes place in the fat body (Table 1). Furthermore, *T*. *rugatulus* may employ additional genes for longevity regulation, compared to the known candidates from model species (Table 1), plus additional candidates might be hidden among the non-annotated differentially expressed contigs. Detailed functional analyses to elucidate their specific function will be necessary in the future.

Only few genes (N = 11) showed the same age-specific expression shifts in the two tissue types, indicating little across-tissue consistency. Among these genes, two may represent housekeeping genes, and none of the eleven genes were linked to longevity. In old, fertile queens, *40 S ribosomal protein S2*, being involved in oogenesis in *D*. *melanogaster*, was consistently upregulated in both tissues, making it a good fertility candidate gene in our species. The general picture indicates a low consistency in expression pattern across tissues, which is in agreement with a recent study on the ant *Lasius niger*^24^, stressing the necessity of conducting tissue-specific expression analyses.

The relative expression level of upregulated genes in old queens was at least twice higher in both tissues compared to upregulated genes in young queens. As gene expression by itself is energy consuming^56^, this finding might be related to established queens having a larger work force, and thus more resources available than founding queens, and may thus be able to invest more in elevated gene expression levels. In line with this hypothesis the co-expression network analysis reveals that young founding queens have a more active lipid catabolism as they utilize more body reserves than the old queens.

The overall variance in expression was higher in older queens compared to young ones, which may be explained by a higher across-sample variance in chronological age among established queens compared to rather similar aged founding queens. On the other hand, we found a much stronger change in gene expression related to worker number in young, compared to old queens. Taken together these results might be due to a stronger effect of worker number on social environment in young queens with few workers (N workers = 0–8, Supplementary Table S1), compared old ones with relatively large established colonies (N workers = 197–337, Supplementary Table S1).

### Fecundity and immunity signatures

Despite significant differences in ovary development, relatively few genes related to fertility were differentially expressed between the highly fecund old queens and the young ones. Indeed, more than five times as many eggs were in development in the ovaries of old queens compared to young ones. Possibly the fat body is the wrong tissue to look for differential expression of fertility genes, which might be more likely to differ in their expression in the ovaries^57^. Another not mutually exclusive hypothesis is that young queens have already activated fertility genes, which are not yet reflected in their ovary development. Consistent with our observations, *Cardiocondyla obscurior* ant queens have been shown to increase their fecundity with age independently of the number of workers in the nest^58^.

In the fat body, young queens activate the TOLL signalling pathway, which plays an important role in insect immunity^51^. This fits to our prediction, and might reflect a higher pathogen exposure during the mating flight and the colony-founding phase. Older queens live in a highly protected environment where pathogen pressure is very low and social immunity strategies of workers shield queens from parasites^59^. An example of plastic regulation of the immune system according to age and environmental conditions (colony age) has been evidenced in bumblebee workers^60^. Immunocompetence is costly and may be traded-off with fecundity and longevity^61–63^. Old queens might thus be able to avoid the costs of upregulating immune genes by delegating the immune defence to workers^64^, and potentially invest these resources into other functions such as body maintenance (e.g. antioxidant production) and in egg production^65^.

### Candidate genes and altered regulation in *T. rugatulus*

We detected a number of candidate genes potentially involved in the reversal trade-off between fertility and longevity in social insect queens (Table 2). In both tissues, old queens upregulated multiple genes involved in longevity pathways in *Drosophila* and mammals. These candidates, such as *hsp70*, *SOD* and *CAT*, play an important role in the reduction of oxidative stress-related molecular damage^54,55^. In *Drosophila* the decrease in stress resistance with age is considered a consequence of an age-related reduction of *hsp70* expression^49,66^. SOD and CAT are known as powerful antioxidants and in *Drosophila* and *C*. *elegans* the overexpression of CAT reduces oxidative stress and extends lifespan^50,62^. Moreover, the expression and the activity of *SOD* and *CAT* is known to decline naturally with age in solitary species including *Drosophila*^47,67,68^, but also in the brain and abdomen of honeybee queens^69^. In *T*. *rugatulus* queens we observe the opposite pattern with stronger expression in old queens. This is similar to termites, where *CAT* is up-regulated in queens compared to workers, which correlates with a lower rate of protein oxidation and may thus be involved in the lifespan differences between the two castes^25^. This finding is corroborated by the enrichment of *stress response to reactive oxygen species* as well as *regulation of response to oxidative stress* mechanisms in fat bodies of old established queens, indicating that established queens activate mechanisms dealing with oxidative stress-related damage commonly associated with aging, suggesting a higher investment into body maintenance^4,25,66^. However a higher investment in body maintenance through an overproduction of antioxidants may not be the only way for living long in every social insects, but maybe species-specific. For example honeybee workers upregulate *CAT* and *SOD* compared to queens^69^ but live shorter. Alternatively investing in molecular repair may also contribute to queen longevity as in the ant *Lasius niger* queens upregulate somatic repair genes rather than antioxidant, compared to workers^70^. Currently, we cannot distinguish whether the higher expression of anti-oxidants in older, established queens is due to a higher age-related generation of ROS or whether it reflects a preventive mechanism independent of ROS level. Oxidative stress measurements of individuals with and without stress (e.g. using paraquat), would allow to identify the cause and effect of the observed age-related increase in stress response genes in *T*. *rugatulus* queens. While our results indicate a higher investment into anti-aging mechanisms in old queens through oxidative stress reduction, the enrichment of the *SAM biosynthetic process* in the fat body of older queens suggests that not all longevity pathways are upregulated to prevent aging in ant queens. In *Drosophila*, the best understood insect aging model, S-adenosylmethonine (SAM), the first metabolite of methionine, increases under a methionine-rich diet which negatively affects lifespan, but positively affects reproduction^71,72^. Moreover, the total amount of SAM in the fat body is associated with aging^71^ while blocking SAM biosynthesis or enhancing SAM degradation extends lifespan, in *Caenorhabditis elegans*^73^ and *Drosophila*^71^, respectively. In *T*. *rugatulus* the high production of SAM could facilitate egg production, whereas the life-shortening effects might be overcome by other somatic maintenance mechanisms, for example through the production of anti-oxidants. This hypothesis could be experimentally tested by sequentially knocking down downstream genes of SAM synthesis, and studying longevity and fecundity and associated gene expression including *CAT* and *SOD*.

Tryptophan (TRY) metabolism and kynurenine (KYN) pathway of TRY degradation are powerful regulators of age-related disorders and lifespan in many organisms^52,74^. In humans and rats an increasing ratio of KYN/TRY is associated with aging and is shown to be a strong predictor of age-related diseases and mortality (Table 2 ^52,75,76^. In *Drosophila*, inhibition of TRY catabolism into KYN extends lifespan, and elevation of *kynurenic acide*, an immediate metabolite of KYN, induces aging^74^. The function *tryptophan catabolism into kynurenine* was enriched in young queens. This is in agreement with our predictions of a lower investment into somatic maintenance in young queens that have a higher extrinsic mortality and other investment priorities. Moreover, those results point again to an opposite age-related expression pattern compared to other solitary organisms that could underlie modification in the KYN and related pathways, specific to our species, and potentially involved in the remodelling of the trade-off between lifespan and reproduction in social insect (Table 2). Alternatively, this finding could also reflect a higher stress level of founding queens leading to the activation of immune defences^75^. Moreover, the existence of other mechanisms involved in the extreme longevity of queens could escape our analysis if their activity remains stable over queen lifetime. Investigating how workers and queens differ in gene expression and how they change the expression of longevity genes with age could allow to identify these mechanisms.

## Conclusions

Gene expression changes in response to age were much more pronounced in the fat body compared to the brain. Among these, we were able to identify a number of longevity candidates, but only few fecundity associated genes and pathways despite strong differences in fecundity between the two queen types. Young queens rather invest in immunity, starvation resistance, and prevention of mitotic replication mistakes despite a physiological rearrangement associated with the founding phase. In contrast, highly fertile, established queens that have a reduced extrinsic mortality rate and the support of many workers, seem to invest more in somatic maintenance mechanisms through the overproduction of antioxidants. This observation suggests a plastic regulation of somatic maintenance in ant queens. Finally, we identify two candidate genes (*CAT* and *SOD*), and one candidate pathway (TRY/KYN pathway), which are potentially involved in the reversed trade-off between lifespan and fecundity in *T*. *rugatulus* queens (Table 2).

## Supplementary information

Supplementary Figures S1 to S6, Supplementary Table S1 and S9 with their respective captations, as well as captation of the Supplementary Tables S2 to S8 and S10

Supplementary Table S2

Supplementary Table S3

Supplementary Table S4

Supplementary Table S5

Supplementary Table S6

Supplementary Table S7

Supplementary Table S8

Supplementary Table S10

## Data Availability

All sequence data for this study as well as assembled contigs, read count, processed data as well as command used were archived at NCBI’s Short Read Archive (SRA) under study accession N° GSE111415.
