# Supplementary material for: Long-lived Temnothorax ant queens switch from investment in immunity to antioxidant production with age
Source: Sci Rep. 2019 May 13;9:7270. doi: 10.1038/s41598-019-43796-1 (PMC6514213; doi:10.1038/s41598-019-43796-1)
Supplement: Supplementary file 1 — Supplementary Figures S1 to S6, Supplementary Table S1 and S9 with their respective captations, as well as captation of the Supplementary Tables S2 to S8 and S10 [file 41598_2019_43796_MOESM1_ESM.pdf]

Supplementary information from:

Long-lived *Temnothorax* ant queens switch from investment in immunity to antioxidant production with age

Matteo Antoine Negroni, Susanne Foitzik, Barbara Feldmeyer

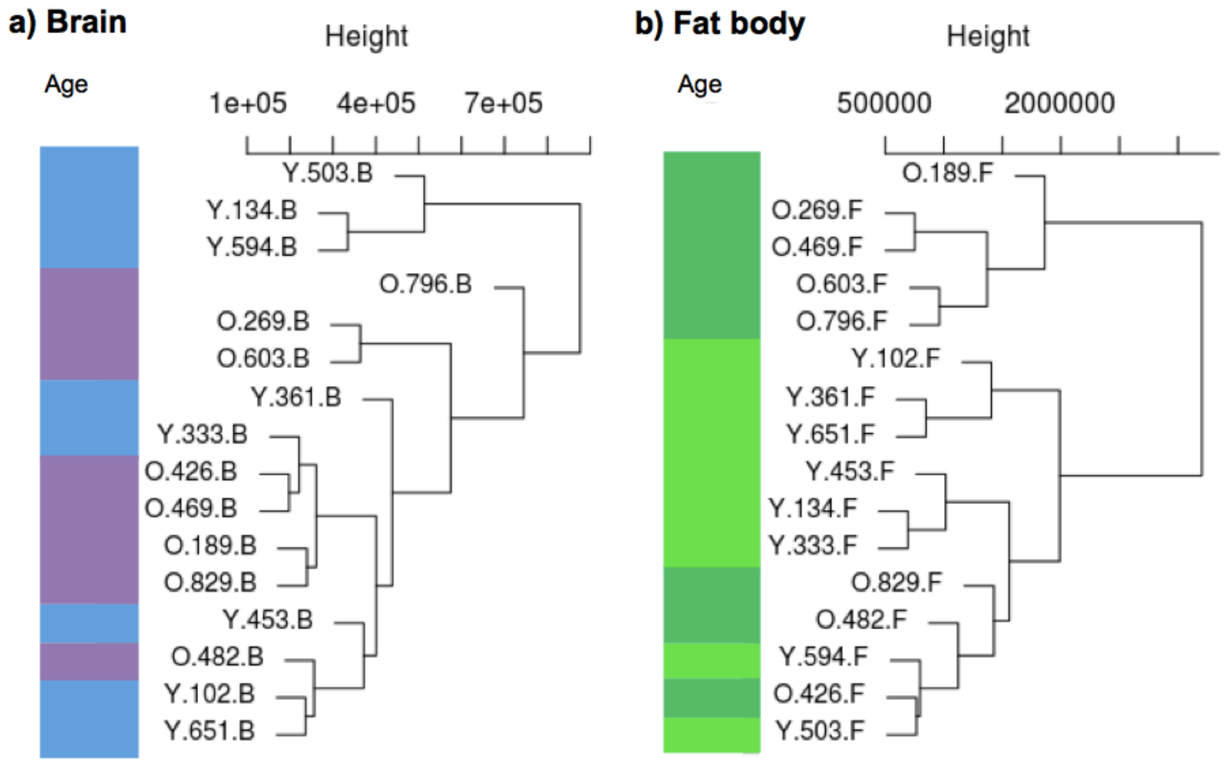

Figure S1: Dendrograms depicting sample similarity per age (Y = young, and O = old queens) based the top 15,000 contigs with the highest variance across samples (a) for the brain and (b) for the fat body.

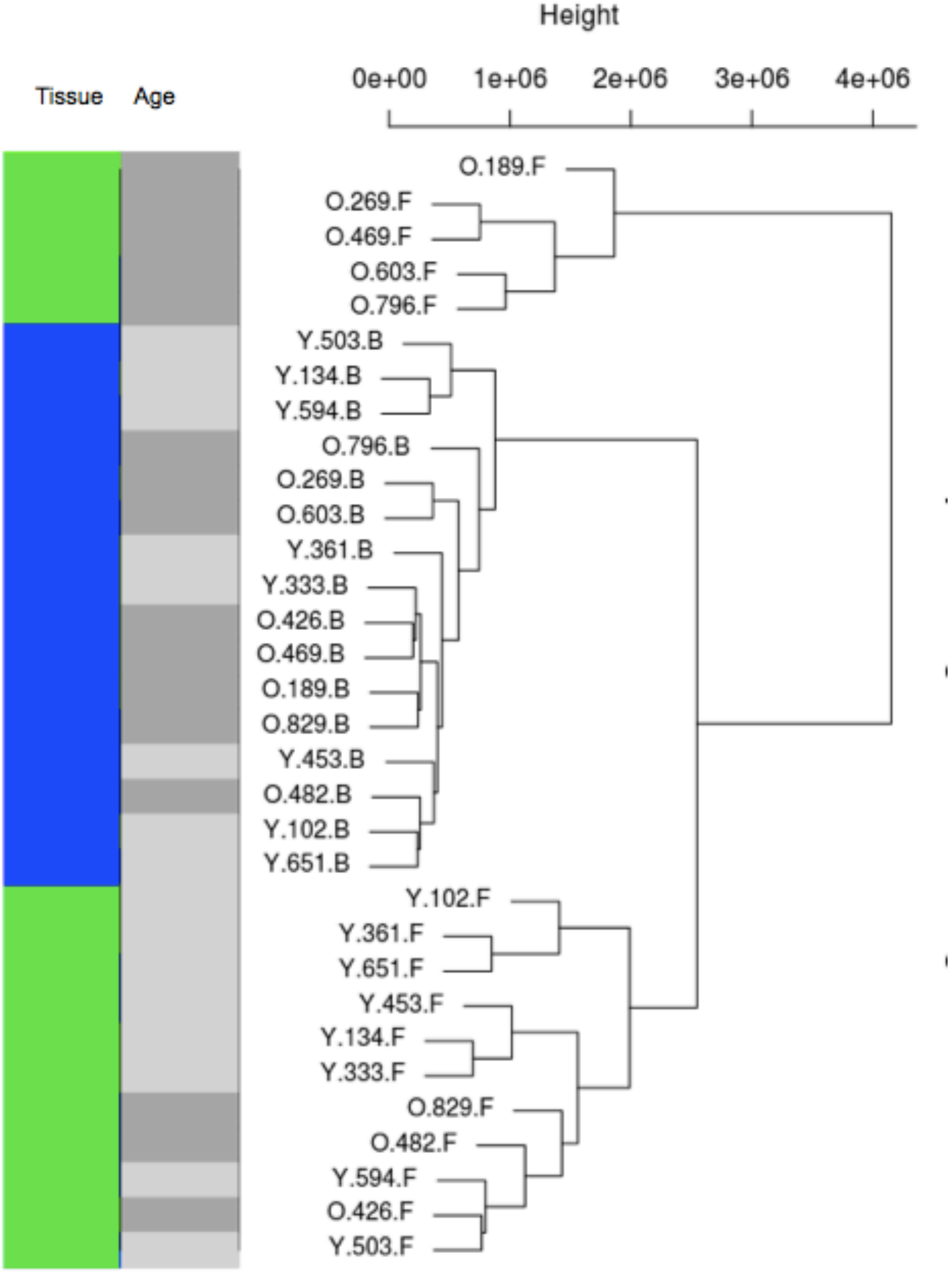

Height
0e+00
1e+06
2e+06
3e+06
4e+06
Tissue
Age
O.189.F
O.269.F
O.469.F
O.603.F
O.796.F
Y.503.B
Y.134.B
Y.594.B
O.796.B
O.269.B
O.603.B
Y.361.B
Y.333.B
O.426.B
O.469.B
O.189.B
O.829.B
Y.453.B
O.482.B
Y.102.B
Y.651.B
Y.102.F
Y.361.F
Y.651.F
Y.453.F
Y.134.F
Y.333.F
O.829.F
O.482.F
Y.594.F
O.426.F
Y.503.F

Figure S2: Dendrogram of sample similarity according to tissue (B = brain or F = fat body) and age (Y = young, and O = old), based on all available contigs. The colours reveal clustering first by tissue (fat body in green, brain in blue) and then by age (young queens light grey, old queens dark grey). Within each tissue samples of old and young queens separate quite well.

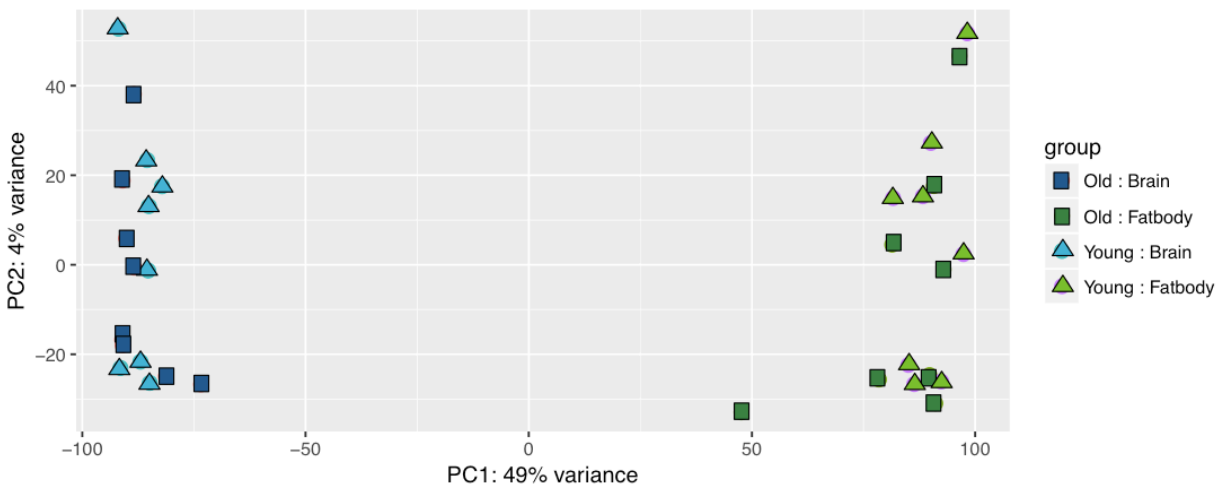

Figure S3: PCA plot based on the standardized read count per contig for all samples. Squares = old queens, triangles = young queens, green = fat body, blue = brain.

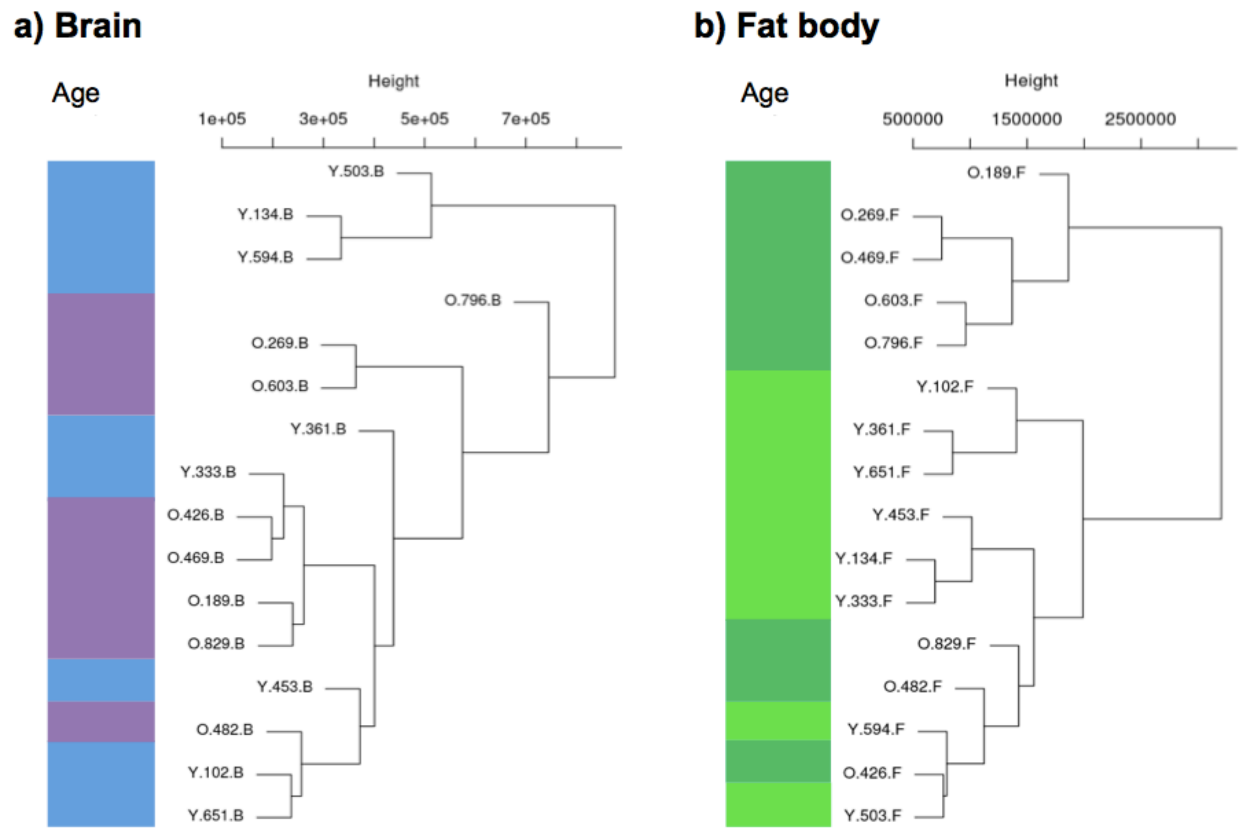

Figure S4: Dendrograms depicting sample similarity based on read counts per contig (a) for the brain and (b) for the fat body. The colours summarize the clustering per age with blue or light green young queens and purple or dark green the old queens respectively.

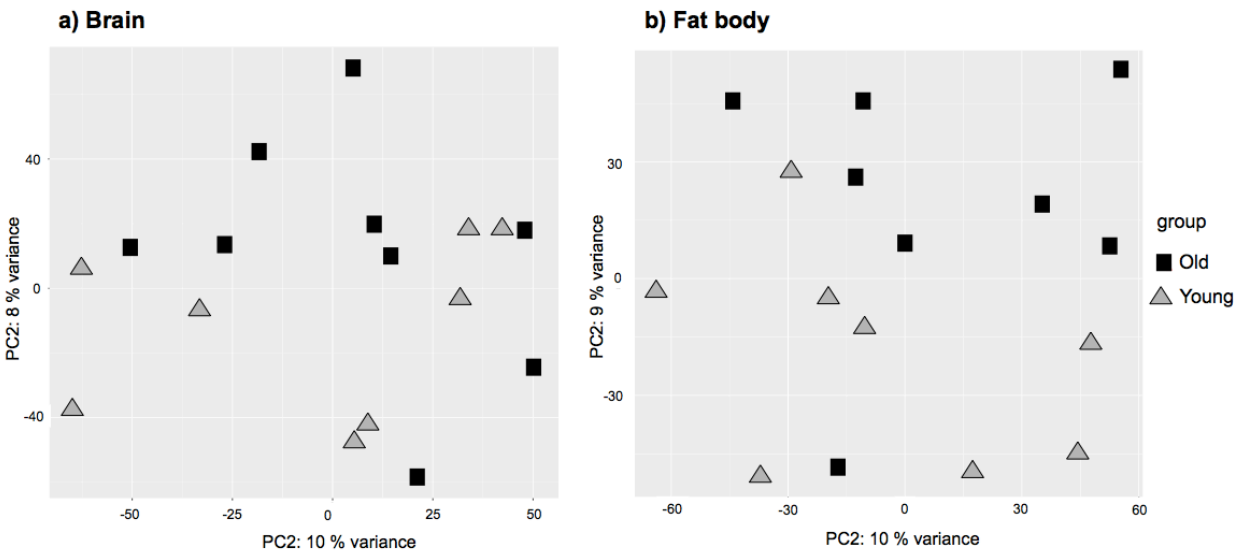

Figure S5: PCA plot based on standardized read counts per contig for each tissue separately: a) brain, and b) fat body. Black squares depict samples of old queens, and grey triangles samples of young queens.

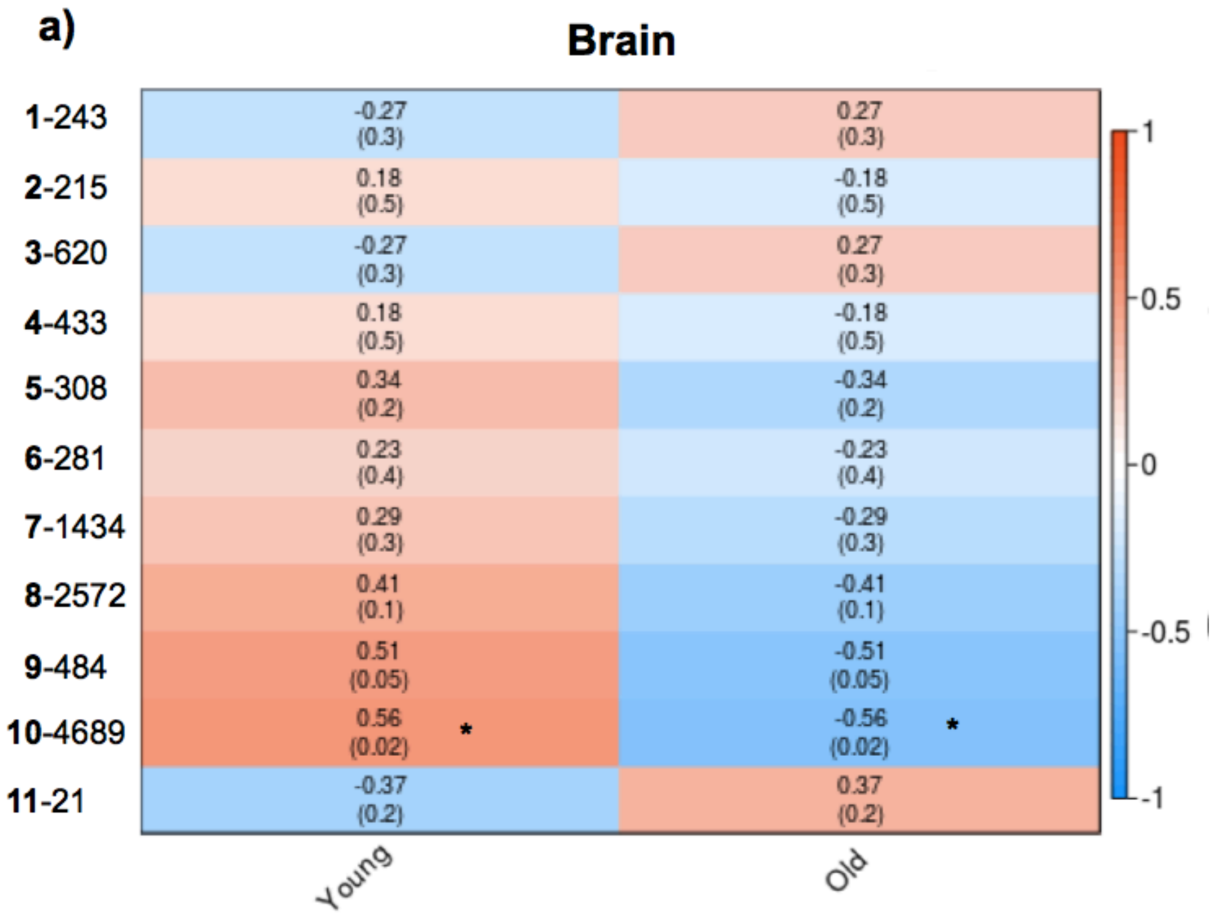
a)
Brain
1-243
2-215
3-620
4-433
5-308
6-281
7-1434
8-2572
9-484
10-4689
11-21
-0.27 (0.3)
0.27 (0.3)
0.18 (0.5)
-0.18 (0.5)
-0.27 (0.3)
0.27 (0.3)
0.18 (0.5)
-0.18 (0.5)
0.34 (0.2)
-0.34 (0.2)
0.23 (0.4)
-0.23 (0.4)
0.29 (0.3)
-0.29 (0.3)
0.41 (0.1)
-0.41 (0.1)
0.51 (0.05)
-0.51 (0.05)
0.56 (0.02) *
-0.56 (0.02) *
-0.37 (0.2)
0.37 (0.2)
Young
Old
1
0.5
0
-0.5
-1

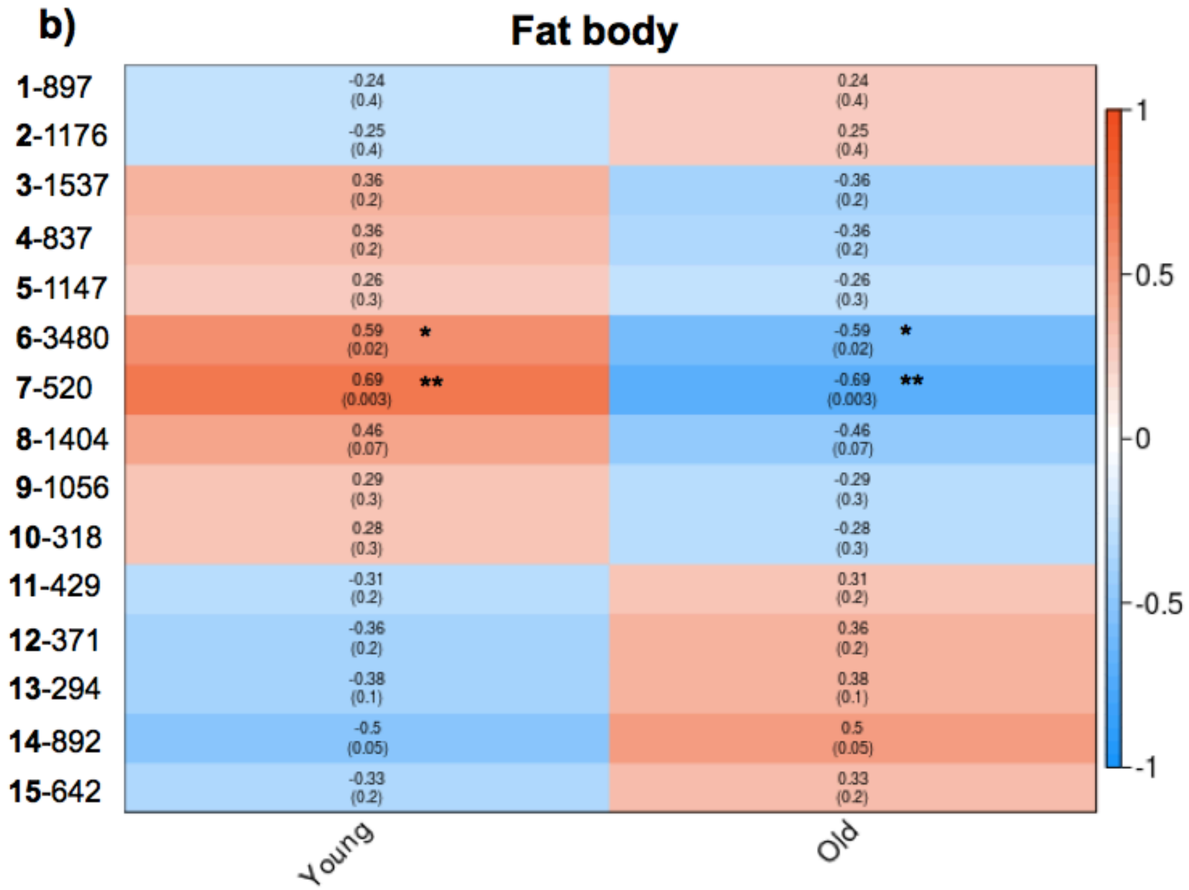

Figure S6: Heat map of the module-traits relationship between old and young queens for the brain (a) and for the fat body (b). The sign and the intensity of the association are represented by colors (positive: red and negative: blue). For each module the coefficient of correlation (on top) as well as the p-value (below in parenthesis, with additional information about significance * and ** at the respective threshold of 0.05 and 0.01) are given. On the left, module numbers (in bold), as well as the number of contigs per module, are listed. Module 11 in the brain and module 15 in the fat body data contain all genes that could not be correlated to genes in any other module.

Table S1: Information on collection sites of *Temnothorax rugatulus* in the Chiricahua Mountains in Arizona and colony composition of queens. We collected ant colonies a a dozen of weeks after the mating flight, so that most founding queens had no or just the first generation of workers. Old queens were sampled from large monogynous field colonies and were thus expected to be several years if not decades old.

Queen ID	Date of collection	Coordinates	Age class	N of workers
102	24/08/15	31.910837; -109.252077	Young	0
134	24/08/15	31.910837; -109.252077	Young	4
333	27/08/15	31.908966; -109.247523	Young	6
361	27/08/15	31.908966; -109.247523	Young	2
503	30/08/15	31.850508; -109.325624	Young	1
594	30/08/15	31.850508; -109.325624	Young	0
651	02/09/15	31.850508; -109.325624	Young	8
453	29/08/15	31.909071; -109.253337	Young	2
189	25/08/15	31.909071; -109.253337	Old	287
269	26/08/15	31.909071; -109.253337	Old	283
426	29/08/15	31.910687; -109.244433	Old	211
469	29/08/15	31.912552; -109.241957	Old	249
482	29/08/15	31.912552; -109.241957	Old	217
603	30/08/15	31.850508; -109.325624	Old	266
796	03/09/15	31.850508; -109.325624	Old	197
829	03/09/15	31.855962; -109.329143	Old	337

Table S2: Summary of the quality estimate comparison of four different assemblies (from the programs *Soap*, *Trinity*, *MIRA* and *Bridger*) conducted using the program *TransRate*.

Supplementary_TableS2.xlsx

Table S3: Results of the functional enrichment analyses based on: the list of young queen brain up-regulated contigs with a unique isoform per gene (a) and young queen fat body up-regulated ones (b) from the between age class comparison per tissue; the list of old queen brain up-regulated contigs with a unique isoform per gene (c) and old queen fat bodies up-regulated ones (d), from between age class comparison per tissue.

Supplementary_TableS3.xlsx

Table S4: List of contigs and annotations from a BlastX search against the invertebrate non-redundant reference database.

Supplementary_TableS4.xlsx

Table S5: Lists of significantly differentially expressed contigs (FDR-p < 0.05) from the within brain, between age class comparison, with: the entire list of differentially expressed contigs (a); old queens up-regulated contigs (b); young queens up-regulated contigs (c); a subset of old queens up-regulated contigs filtered for a Log2FoldChange > 2 and *UniProt* annotation (d); and a subset of young queens up-regulated contigs filtered for a Log2FoldChange > 2 and *UniProt* annotation (e) old queens up-regulated contigs also upregulated from the within fat body, between age class comparison, with result from the checking for housekeeping genes (f); young queens up-regulated contigs also upregulated from the within fat body, between age class comparison, with result from the checking for housekeeping genes (g).

Supplementary_TableS5.xlsx

Table S6: Lists of significantly differentially expressed contigs (FDR-p < 0.05) from the within fat body, between age class comparison, with: the entire list of differentially expressed contigs (a); old queens up-regulated contigs (b); young queens up-regulated contigs (c); a subset old queens up-regulated contigs filtered for a Log2FoldChange > 2 and *UniProt* annotation (d); and a subset of young queens up-regulated contigs filtered for a Log2FoldChange > 2 and *UniProt* annotation (e); old queens up-regulated contigs also upregulated from the within brain, between age class comparison, with result from the checking for housekeeping genes (f); young queens up-regulated contigs also upregulated from the within brain, between age class comparison, with result from the checking for housekeeping genes (g).

Supplementary_TableS6.xlsx

Table S7: Results of the pathway analyses based on: the list of old queen fat bodies up-regulated contigs (a) and young queen fat bodies up-regulated ones (b) from the within fat body, between age class comparison; the list of old queen brains up-regulated contigs (c) and young queen brains up-regulated ones (d) from the within brain, between age class comparison.

Supplementary_TableS7.xlsx

Table S8: Results of the functional enrichment analysis based on: the list of young queen brain up-regulated contigs (a) and old queen brain up-regulated ones (b) from the within brain, between age class comparison; the list of young queen fat body up-regulated contigs (c) and old queen fat body up-regulated ones (d) from the within fat body, between age class comparison.

Supplementary_TableS8.xlsx

Table S9: Summary of the results from the functional enrichment of upregulated contigs per age class for brain and fat body. Significantly enriched functions are indicated in bold for the most relevant ones, and in grey if functions are relevant but only marginally enriched (p-value > 0.05). The indicated p-value was calculated with a Fisher exact test.

Age	Tissue	GO ID	Biological Process	P-value
Young Queens	Fat Body	GO:0046416	D-amino acid metabolic process	**< 0.0001**
		**GO:0006367**	**transcription initiation from RNA pol. II promoter**	**0.002**
		**GO:0000398**	**mRNA splicing, via spliceosome**	**0.004**
		GO:0043504	mitochondrial DNA repair	0.007
		**GO:0006351**	**transcription, DNA-templated**	**0.011**
		**GO:0006499**	**N-terminal protein myristoylation**	**0.013**
		**GO:0006482**	**protein demethylation**	**0.013**
		GO:0046294	formaldehyde catabolic process	0.013
		GO:0000387	spliceosomal snRNP assembly	0.017
		**GO:0006402**	**mRNA catabolic process**	**0.020**
		**GO:0008063**	**Toll signaling pathway**	**0.020**
		GO:0048011	neurotrophin TRK receptor signaling pathway	0.020
		GO:0006278	RNA-dependent DNA biosynthetic process	0.020
		**GO:0030163**	**protein catabolic process**	**0.028**
		**GO:0051603**	**proteolysis involved in cellular proteins**	**0.032**
		**GO:0006479**	**protein methylation**	**0.032**
		**GO:0006401**	**RNA catabolic process**	**0.033**
		**GO:0019441**	**tryptophan catabolic process to kynurenine**	**0.033**
		**GO:0007064**	**mitotic sister chromatid cohesion**	**0.033**
		**GO:0035176**	**social behavior**	**0.033**
		**GO:0006412**	**translation**	**0.033**
		**GO:0016567**	**protein ubiquitination**	**0.037**
		**GO:0032007**	**negative regulation of TOR signalling**	**0.039**
		**GO:0007094**	**mitotic spindle assembly checkpoint**	**0.039**
		**GO:0043066**	**negative regulation of apoptotic process**	**0.046**
		**GO:0000122**	**negative regulation of transcription from RNA pol. II promotor**	**0.046**
		**GO:0006338**	**chromatin remodeling**	**0.05**
	Brain	GO:0055114	oxidation-reduction process	0.0095
Old Queens	Fat body	**GO:0006412**	**translation**	**< 0.0001**
		GO:0015986	ATP synthesis coupled proton transport	0.003
		**GO:0006556**	**S-adenosylmethionine biosynthetic process**	**0.003**
		**GO:0006979**	**response to oxidative stress**	**0.006**
		GO:0009082	branched-chain amino acid biosynthetic process	0.007
		**GO:0045901**	**positive regulation of translational elongation**	**0.007**
		**GO:0045905**	**positive regulation of translational termination**	**0.007**
		**GO:0006452**	**translational frame-shifting**	**0.007**
		GO:0006754	ATP biosynthetic process	0.010
		**GO:0006084**	**acetyl-CoA metabolic process**	**0.016**
		GO:0019878	lysine biosynthetic process via aminoadipic acid	0.021
		GO:0006685	sphingomyelin catabolic process	0.021
		**GO:0006414**	**translational elongation**	**0.031**
		GO:0006099	tricarboxylic acid cycle	0.036
		GO:0003333	amino acid transmembrane transport	0.038
		**GO:1901031**	**regulation of response to reactive oxygen species**	**0.041**
		GO:0006075	(1->3)-beta-D-glucan biosynthetic process	0.041
		**GO:0006897**	**endocytosis**	**0.07**
	Brain	GO:0006412	translation	**< 0.0001**
		GO:0006596	polyamine biosynthetic process	0.003
		GO:0006075	(1->3)-beta-D-glucan biosynthetic process	0.008
		GO:0046168	glycerol-3-phosphate catabolic process	0.026
		GO:0006084	acetyl-CoA metabolic process	0.038
		GO:0006096	glycolytic process	0.042

Table S10: Results on enriched functions per module for (a) the brain and (b) the fat body, following the WGCNA and complementary to Figure S6.

Supplementary_TableS10.xlsx
